# Telbivudine Reduces Parvovirus B19-Induced Apoptosis in Circulating Angiogenic Cells

**DOI:** 10.3390/v11030227

**Published:** 2019-03-06

**Authors:** Thomas Zobel, C.-Thomas Bock, Uwe Kühl, Maria Rohde, Dirk Lassner, Heinz-Peter Schultheiss, Caroline Schmidt-Lucke

**Affiliations:** 1 Department of Cardiology and Pneumology, Charité-Universitätsmedizin Berlin, 10117 Berlin, Germany; Thomas.Zobel@Verwaltung.Uni-Muenchen.de (T.Z.); uwe.kuhl@charite.de (U.K.); heinz-peter.schultheiss@charite.de (H.-P.S.); 2 Department of Infectious Diseases, Robert-Koch-Institut, 13353 Berlin, Germany; bockc@rki.de; 3 Institut für Kardiale Diagnostik und Therapie (IKDT), 12203 Berlin, Germany; maria.rohde@ikdt.de (M.R.); info@ikdt.de (D.L.); 4 MEDIACC GmbH, 10713 Berlin, Germany; 5 Berlin-Brandenburg Centre for Regenerative Therapies, 13353 Berlin, Germany

**Keywords:** telbivudine, B19V, circulating angiogenic cells, apoptosis, caspase-3, BIRC3 (cIAP-2)

## Abstract

**Aims:** Human parvovirus B19 (B19V) infection directly induces apoptosis and modulates CXCR4 expression of infected marrow-derived circulating angiogenic cells (CACs). This leads to dysfunctional endogenous vascular repair. Treatment for B19V-associated disease is restricted to symptomatic treatment. Telbivudine, a thymidine analogue, established in antiviral treatment for chronic hepatitis B, modulates pathways that might influence induction of apoptosis. Therefore, we tested the hypothesis of whether telbivudine influences B19V-induced apoptosis of CAC. **Methods and Results:** Pretreatment of two CAC-lines, early outgrowth endothelial progenitor cells (eo-EPC) and endothelial colony-forming cells (ECFC) with telbivudine before in vitro infection with B19V significantly reduced active caspase-3 protein expression (−39% and −40%, both *p* < 0.005). Expression of Baculoviral Inhibitor of apoptosis Repeat-Containing protein 3 (BIRC3) was significantly downregulated by in vitro B19V infection in ECFC measured by qRT-PCR. BIRC3 downregulation was abrogated with telbivudine pretreatment (*p* < 0.001). This was confirmed by single gene PCR (*p* = 0.017) and Western blot analysis. In contrast, the missing effect of B19V on angiogenic gene expression postulates a post-transcriptional modulation of CXCR4. **Conclusions:** We for the first time show a treatment approach to reduce B19V-induced apoptosis. Telbivudine reverses B19V-induced dysregulation of BIRC3, thus, intervening in the apoptosis pathway and protecting susceptible cells from cell death. This approach could lead to an effective B19V treatment to reduce B19V-related disease.

## 1. Introduction

Human parvovirus B19 (B19V), belonging to the genus Erythrovirus of the Parvoviridae family, is a single-stranded DNA virus responsible for a wide range of clinical manifestations. Although the majority of clinical disorders are generally self-limiting and subclinical [1], in certain cases, the disease may turn chronic and clinically relevant. The latter may result from either direct virus-mediated injury, increased apoptosis [2], inadequacy of the specific anti-viral immune response [3] or dysregulated trafficking of cells involved in endothelial regeneration [4].

B19V replication and expression profiles highlight the very restricted cellular targets defined by a specific receptor status, erythroid lineage, and differentiation stage [2,5,6,7,8]. Recently, we have identified different cell types belonging to the heterogenous group of marrow-derived circulating angiogenic cells (CACs) with similarities to the erythroid and endothelial lineage, to be targets for B19 infection [2]. CAC play a key role in cardiovascular regeneration [9]. B19V directly induces apoptosis of CAC through the viral proteins NS1, VP1 [2], and the small 11kDa protein and impairs their trafficking [4]. Among CAC, the so-called early outgrowth endothelial progenitor cells (eo-EPC) have been shown to parallel disease progression in atherosclerosis [10]. Clonally distinct endothelial colony-forming cells (ECFC) with high proliferative potential expressing endothelial cell surface antigens form robust vascular structures in vivo and in vitro [11,12] and are associated with improved cardiac function [13].

Inhibitor of Apoptosis (IAP) gene products play an evolutionarily conserved role in regulating programmed cell death in diverse species [14]. The Baculoviral Inhibitor of apoptosis Repeat-Containing protein 3 (BIRC3; cellular inhibitor of apoptosis-2, cIAP-2) is a member of the Inhibitor of Apoptosis family [15,16] that inhibit apoptosis by interfering with the activation of caspases-3, -6, and -7 [14,17,18], including the effector caspase-9 [14] and caspase-8 [15,19]. We have previously shown the induction of apoptosis in CAC through activation of caspases-8 and -10 [2]. Expression of BIRC3 has been strongly linked to cell survival in virally-associated cancer and been classified as an oncogene [20]. Furthermore, BIRC3 was found to inhibit hepatitis B virus (HBV) protein synthesis, viral replication, and transcription [21,22] and ubiquitination of cellular factors essential for antiviral response [23].

The synthetic thymidine nucleoside analogue telbivudine, approved for the treatment of hepatitis B [24,25], inhibits DNA dependent second strand DNA synthesis [24,25]. Mechanistically, telbivudine-5′-triphosphate is incorporated into the nascent HBV DNA strand by the HBV DNA polymerase, competing with the natural substrate thymidine-5′-triphosphate, finally leading to chain termination. Furthermore, telbivudine modulates expression of the cytokines TNFα and Interferon-gamma (INFγ) [26,27,28] and influences NF-κB level [29] restoring cellular immune response. A direct effect of telbivudine on apoptosis has so far never been investigated.

Therefore, we tested the hypothesis of telbivudine influencing B19V-induced apoptosis of CAC We, therefore, analysed potential signalling pathway of apoptosis induction by B19V and the effect of telbivudine treatment in vitro.

## 2. Methods

### 2.1. Cell lines and Isolation of Primary Cells

Mononuclear cells (MNC) were isolated by density-gradient centrifugation with Biocoll (Biochrom) from peripheral blood of healthy donors. Cultivation of eo-EPC was performed as described previously [2], using the same seronegative male donors for corresponding experiments.

Human umbilical cord blood ECFC were purchased from Lonza and cultivated as recommended by the manufacturer. Experiments with ECFC were conducted at passage 5 to 7 since these yielded optimal results [2]. All in vitro experiments were conducted at least in triplicate. Cells were cultivated at 37 °C, 5% CO_2_.

For the rationale to use different cell lines for the individual experiments, please see [2].

### 2.2. Telbivudine Treatment of Cell Cultures

Telbivudine (sc-222340, Santa Cruz Biotechnology) was dissolved in water and added at final concentrations of 10 ng/mL (41.3 nM) to the respective cell culture medium 2 h before infection.

### 2.3. Virus Stock and Infection

B19V belonging to genotype 1 was purified from the serum of a patient with B19V as described previously [2]. The concentration of virus particles was quantified by real-time PCR by means of B19V genome equivalents (GE). Uninfected, virus-free control plasma was purified using identical experimental conditions. Until otherwise indicated, cells were infected in the appropriate growth medium with 3000 GE/cell or treated with equal volumes of purified control plasma. As an internal control for successful B19V infection, detection of B19V DNA and RNA by nested PCR was performed as described previously with primers specific for the NS1 coding sequence [2] before performing the following experiments.

Consequently, 4 different experimental settings were compared: (a) plasma treatment, no telbivudine (plasma, no telbivudine); (b) plasma treatment, telbivudine (plasma, telbivudine); (c) B19V-infected plasma, no telbivudine (B19V, no telbivudine); (d) B19V-infected plasma, telbivudine (B19V, telbivudine).

### 2.4. RNA Isolation and Reverse Transcriptase PCR

Total RNA isolation with the RNeasy Kit (Qiagen, Hilden, Germany), DNase digestion of purified RNA with Turbo DNA-free DNase (Life Technologies, Waltham, Massachusetts, U.S.) and cDNA synthesis with M-MLV Reverse Transcriptase (RT) RNase H minus (Promega, Fitchburg, Wisconsin, USA) were performed according to the manufacturer’s instructions. Turbo DNA-free digestion (Thermo Fisher Scientific, Waltham, Massachusetts, USA) was performed with 500 ng to 1.5 µg total RNA as recommended. At least 300 ng DNase-digested RNA was reverse transcribed with M-MLV RT as +RT reactions versus equal amounts of RNA under the same conditions without M-MLV RT as –RT reactions. cDNA synthesis was performed with oligo(dT) primers (Thermo Fisher Scientific).

### 2.5. Fluorescence Associated Cell Sorting (FACS) Analysis for Active Caspase-3

Active caspase-3 staining was performed with infected CAC as described previously [2]. CACs were harvested with Accutase (Thermo Fisher Scientific), fixed with 4% paraformaldehyde and washed. CACs were incubated for 15 min. at 4 °C with 2% human FcR-blocking reagent and washed once with phosphate buffered saline (PBS) (Sigma Aldrich, St. Louis, Missouri, USA). Afterwards, cells were permeabilized for intracellular caspase-3 staining with Cytofix/Cytoperm-solution (BD Cytofix/Cytoperm Permeabilization Kit, BD Biosciences) for 15 min. at 4 °C. Cells were resuspended in 100 µL BD Perm/Wash buffer and incubated with V450 rabbit anti-active caspase-3 antibody (1:33 diluted, BD Biosciences, Erembodegem, Belgium) for 20 min. at 4 °C in the dark. After the incubation, cells were washed once with BD Perm/Wash buffer and resuspended in PBS. FACS analyses were done on a BD FACSCanto II (BD Biosciences).

### 2.6. PCR Array for Human Apoptosis and Angiogenesis

A Human Apoptosis (PAHS-12Z) and Angiogenesis (PAHS-024Z) RT^2^ Profiler PCR Array was performed with the RT² First Strand Kit and the RT² SYBR Green/ ROX Master Mix according to the manufacturer’s instructions (Qiagen) using 500 ng input RNA. Data were analysed with the PCR Array Data Analysis Software (Qiagen). For this, only ECFC were used since this cell type can be cultivated in greater quantities independent of intersubject variability and expected confounders through the experimental manipulations.

### 2.7. BIRC3 Single PCR Levels

Quantitative PCR for BIRC 3 was performed using 50 ng cDNA with the Fast Blue qPCR MasterMix plus ROX (Eurogentec, Seraing, Belgium) and the BIRC3 TaqMan GeneExpression Assay (HS00985031_g1) according to the manufacturer’s instructions. Standardization was performed with a primer/probe set against ribosomal Protein S29 (RPS29) using a primer and probe set designed by the Roche Universal Probe Library (UPL) Assay Design Center and using Universal Probe number 25 using the Fast Blue qPCR MasterMix plus ROX and 50 ng cDNA according to the manufacturer’s instructions.

### 2.8. Statistical Analysis

All experiments except for the ones for the PCR-arrays (for apoptosis (n = 2) and for angiogenetic genes, n = 1) were performed as at least three independent experiments. Continuous variables were tested for normal distribution with the Kolmogorov–Smirnov test. Non-normally distributed continuous variables were compared by the Mann–Whitney-U test for two groups or by the Kruskal–Wallis test with more than two subgroups with post hoc analysis (Wilcoxon). Data are expressed as mean ± SD, unless otherwise stated. Comparison of categorical variables was generated by the Pearson χ2 test. Statistical significance was assumed if a null hypothesis could be rejected at *p* ≤ 0.05. All statistical analysis was performed with SPSS 21.0 (SPSS, IBM, Armonk, New York, USA.).

## 3. Results

### Telbivudine Inhibits Activation of B19V-Induced Apoptosis through Stabilisation of BIRC3 Levels

**Figure 1:**
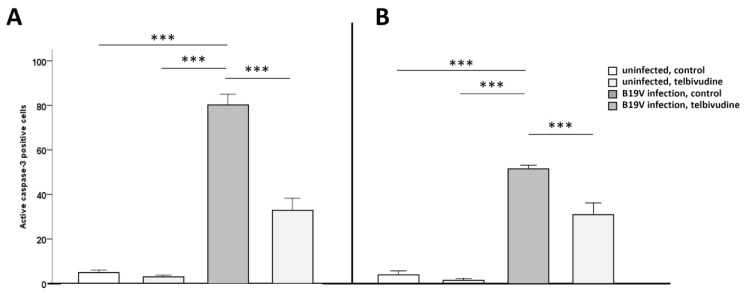
Reduced human parvovirus B19 (B19V)-induced apoptosis after telbivudine pre-treatment in B19V-infected early outgrowth endothelial progenitor cells (eo-EPC) and endothelial colony-forming cells (ECFC). Quantification of active caspase-3 positive in (**A**) ECFC and (**B**) eo-EPC. Data are means +SD, *** is *p* < 0.001.

We have previously shown a strong induction of apoptosis in B19V-infected CAC [2]. Here, we assessed the magnitude of apoptosis in CAC after in vitro infection with B19V and the effect of pre-treatment with telbivudine, respectively. Pre-treatment with telbivudine significantly (*p* < 0.001) reduced B19V-induced apoptosis by 39%, as measured by activation of the effector caspase-3 in ECFC (B19V, no telbivudine 81.5 ± 11.7% vs. B19V, telbivudine 35.8 ± 19.3% active caspase-3 positive cells compared to plasma, no telbivudine 3.3 ± 2.4% vs. plasma, telbivudine 1.8 ± 1.3%; Figure 1A) and eo-EPC by 40%, (*p* < 0.005; B19V, no telbivudine 51.8 ± 2.3% vs. B19V, telbivudine 31.0 ± 5.6% active caspase-3 positive cells compared to plasma, no telbivudine 4.1 ± 1.7% vs. plasma, telbivudine 1.6 ± 0.4%; Figure 1B).

**Figure 2:**
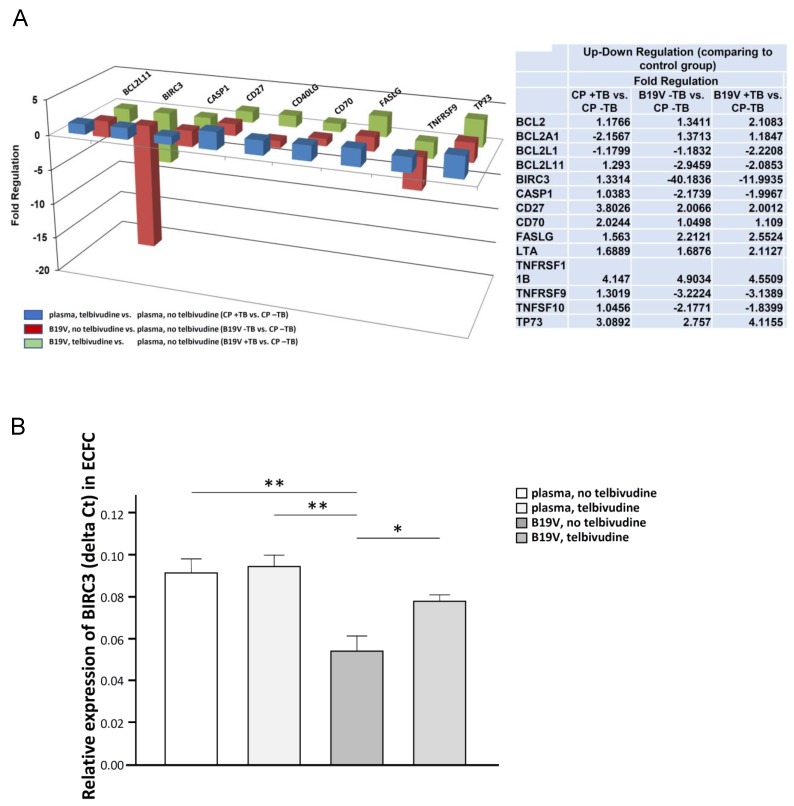
PCR Array for apoptosis and Baculoviral Inhibitor of apoptosis Repeat-Containing protein 3 (BIRC3) after telbivudine pre-treatment in B19V-infected ECFC. (**A**) Regulation of apoptotic genes in ECFC; plasma, telbivudine versus plasma, no telbivudine (blue bars), B19V, no telbivudine versus plasma, no telbivudine (red bars), and B19V, telbivudine versus plasma, no telbivudine (green bars). (**B**) Quantification of BIRC RNA in ECFC, * is *p* < 0.05, ** is *p* < 0.01.

To further dissect the underlying pathway, we analyzed multiple pathways through a PCR array for human apoptosis (*n* = 1) using ECFC. Infection with B19V led to strong downregulation of BIRC3 mRNA levels. Treatment of CAC with telbivudine before B19V infection reversed this effect (Figure 2A). This was confirmed by Single PCR (*n* = 4, Figure 2B), that showed BIRC3 levels to be less downregulated when ECFC were pretreated with elbivudine before B19V infection.

Having shown that B19V infection affects trafficking of CAC [4], we subsequently analysed the effect of telbivudine on angiogenic genes with an RT2 Profiler PCR array analysis for angiogenic genes (n = 1). We saw very little changes in a few other genes, so that we postulate a post-transcriptional effect of B19V on CXCR4.

## 4. Discussion

The results of this study show for the first time an anti-apoptotic effect of the nucleoside analogue telbivudine through normalisation of BIRC3 levels in B19V-induced apoptosis in CACs, thus, providing a novel approach to protecting cells from B19V damage. These results extend the knowledge of activities of telbivudine against known human viruses. Thus, telbivudine may be an interesting candidate to unravel new insights into cellular mechanisms in B19V infection.

B19V infection is restricted to a small number of cell lines including erythroid progenitor cells, erythroblastoid cell line UT7/Epo-S1 [6,7] or CAC [2], the latter are important players in endogenous cardiovascular regeneration [30]. B19V infection in humans is associated with impaired endothelial regeneration through induction of apoptosis and dysregulated trafficking of infected CAC [2,4]. Therapy with interferon beta suppresses B19V replication (by 63%) and increases the viability of these erythroid progenitors [31]. However, an effective treatment option for B19V-associated disease is not available.

We, therefore, tested the effect of telbivudine, a thymidine analogon and therapeutic agent well established for treatment of chronic hepatitis B, in B19V infection of CAC focussing on apoptosis and trafficking. Reproducing previous results, B19V infection strongly induced apoptosis in CAC [2], and as demonstrated in this study, B19V-induced apoptosis is mediated through downregulation of the antiapoptotic inhibitors of apoptosis BIRC3 (cellular inhibitor of apoptosis-2 (cIAP2)), a potent suppressor of apoptotic cell death. With pretreatment of telbivudine, downregulation of the anti-apoptotic BIRC3 in CAC is reduced and apoptosis prevented. BIRC3 prevents the proteolytic processing of pro-caspases -3, -6 and -7 by blocking the cytochrome c-induced activation of pro-caspase-9 [14]. Whereas caspase-8 induced proteolytic activation of pro-caspase-3 is not inhibited by BIRC3 [14], the formation of a complex with the TNF-receptor associated factor 1 (TRAF1), TRAF2, and the TNFα receptor (TNFR), activating caspase-8, is hindered [15,19,32]. The direct anti-apoptotic effect of telbivudine in B19V-induced apoptosis could be shown on transcriptional levels of BIRC3. Further research on the precise pathway, on protein levels, up- and downstream signalling and to identify influencing player, i.e., RIP1, TRAF1, TRAF2, TRAIL, TNFR, caspase-8, caspase-6/7/9, is needed [33]. In erythroid progenitor cells, INFγ delays apoptosis, related to the expression of Bcl-x without the involvement of Fas [34]. The TNFα -inducible gene cIAP2 inhibits HBV protein synthesis, viral replication, and transcription [21]. Thus, since telbivudine alters the NF-κB level [29] and modulates expression of the cytokines TNFα and INFγ [26,27,28], the INFγ-mediated apoptosis pathways including NF-κB expression offer a further promising target [35,36,37].

Much to our surprise, no angiogenic gene modulations were detected, postulating a post-transcriptional effect of B19V on CXCR4 that needs to be further dissected.

The identification of compounds active against B19V-induced vascular damage might add therapeutic options to the treatment of B19 infection which, up to now, relies entirely on symptomatic treatment. It is tempting to speculate that the reduction of apoptosis of CAC by telbivudine could improve cell survival, thus, reducing the turn-over rate of CAC, which in turn could preserve the presumably finite pool of regenerative cells. This can be expected to preserve endothelial regenerative function. Whether our preliminary findings can be extended to a specific treatment in B19V-induced diseases in vivo, will have to be evaluated in detail in confirmatory in vitro and in vivo experimental and clinical studies. Furthermore, experiments to show the effect on apoptotic pathways apart from directly interfering with B19V replication are needed. Of interest, the results of these studies might have implications for angiogenesis related to tumour angiogenesis. The research on the role of B19V or its compounds might open new therapeutic approaches.

## Abbreviations

B19V	human parvovirus B19
BIRC3	baculoviral inhibitor of apoptosis repeat-containing protein 3
CAC	circulating angiogenic cells
cIAP-2	cellular inhibitor of apoptosis-2
eo-EPC	early outgrowth endothelial progenitor cells
ECFC	endothelial colony forming cells
FACS	fluorescence associated cell sorting
GE	genome equivalents
HBV	hepatitis B virus
IAP	inhibitor of apoptosis
KDR	kinase insert domain receptor
MNC	mononuclear cells
PBS	phosphate buffered saline
RT	reverse transcriptase
RIP1	receptor interacting protein (RIP) kinases 1
TNF	tumor necrosis factor
TNFR	TNFα receptor
TRAF1	TNF receptor associated factor 1
TRAF2	TNF receptor associated factor 2
TRAIL	TNF-related apoptosis-inducing ligand
